# Controlled Synthesis of BaYF_5_:Er^3+^, Yb^3+^ with Different Morphology for the Enhancement of Upconversion Luminescence

**DOI:** 10.1186/s11671-017-2390-4

**Published:** 2017-12-19

**Authors:** Jialiang Yao, Fenghua Zhao, Chunyang Pan, Jianle Zhuang

**Affiliations:** 1 School of Light Industry and Chemical Engineering, Guangdong University of Technology, Guangzhou, 510006 China; 2 College of Materials and Energy, South China Agricultural University, Guangzhou, 510642 China

**Keywords:** Upconversion, Er^3+^/Yb^3+^ codoped, Luminescent properties, BaYF_5_

## Abstract

In this work, Er^3+^/Yb^3+^-codoped BaYF_5_ with different sizes and shapes have been synthesized by a simple solvothermal method. By changing the fluoride source, pH value, solvent, surfactants, Yb^3+^ concentration, temperature, and reaction time, the optimum synthetic conditions of BaYF_5_:Er^3+^, Yb^3+^ were found to improve the upconversion luminescent properties. It is found that the emission intensity of green and red light is enhanced for several times by the way of using NaBF_4_ as a fluoride source with the comparison of NH_4_F and NaF. Moreover, the effects of different surfactants are not the same. Adding 5% polyetherimide (PEI) as surfactant can also improve the upconversion emission. On the contrary, when sodium citrate (CIT) as another surfactant was used to add, the sizes of the nanocrystals were gradually increased and the luminous properties also declined.

## Background

In recent year, upconversion nanophosphors (UCNPs) have attracted increasing attention due to their use in many fields such as solid state laser devices, fluorescent probe imaging, bioapplication, stereoscopic three-dimensional display, infrared quantum counter, temperature sensor, and anti-fake [1–11]. UCNPs are usually composed of matrix material, activator, and sensitizer [12]. Because of its low phonon energy and excellent chemical stability, fluorides are often used as matrix materials for the preparation of UCNPs. NaYF_4_ [13] nanoparticles with good upconversion emission have a hexagonal phase structure, while the cubic phase results in poor upconversion emission. Recently, some of the UC materials based on BREF_5_ (B = Mg, Ba, Ca, Sr) have also been studied and these newly developed crystals were found to be suitable for UC applications [14, 15]. Er^3+^-doped BaYF_5_ extremely exhibits the strong UC luminescence ability. The luminescence intensity of Er^3+^-doped BaYF_5_ is eight times that of Er^3+^-doped LaF_3_ [16]. When Er^3+^ is used as activator, Yb^3+^ is a representative UC luminescence sensitizer due to their efficient energy transfer [17–21]. Moreover, the charge sizes of Er^3+^ and Y^3+^ match, and their radii are similar (Er^3+^ radius is 0.1 nm, Y^3+^ radius is 0.101 nm) [22]. Therefore, BaYF_5_ is deemed to be an appropriate host for Er^3+^ ions.

The main factors affecting the luminescence properties are particle size, morphology, structure, and others [23, 24]. In order to obtain UC luminescent materials with high efficiency, the controlled synthesis of the spherical particles with suitable size is beneficial to achieve high accumulation of density and scatter light. In this work, samples of Yb^3+^/Er^3+^-codoped BaYF_5_ are fabricated by a solvothermal method. Under the different reaction conditions, the samples with different morphologies and properties were synthesized. NaBF_4_ as fluoride source has a higher UC luminous intensity relative to NH_4_F and NaF. Perhaps it can slowly release F^−^; thus, it is more conducive to make crystal growth and promote UC luminescence. Furthermore, the influence of solvent, surfactants, Yb^3+^ concentration, pH of initial solution, temperature, and reaction time was also reported. Between UC luminous efficiency and various reaction conditions, the regularity and mechanism have been investigated.

## Experimental

All the chemicals are analytical grade, such as Ba(OH)_2_·xH_2_O, Y(NO_3_)_3_·6H_2_O, Yb_2_O_3_, (CH_3_CO_2_)_3_Er, NaBF_4_, NH_4_F, NaF, oleic acid, and HNO_3_, and absolute ethanol was used. Deionized water was used throughout. All chemical materials were used as received without further purification.

### Preparation of Synthetic BaYF_5_:Er^3+^, Yb^3+^

Yb_2_O_3_ was dissolved in dilute HNO_3_ by heating the solution in order to gain the Yb(NO_3_)_3_ solution. In a typical synthetic route, Ba(OH)_2_·xH_2_O, Y(NO_3_)_3_·6H_2_O, (CH_3_CO_2_)_3_Er, and NaBF_4_ were separately dissolved in deionized water. According to the ratio of BaY_1-x-y_F_5_:xEr^3+^, yYb^3+^, the solution of Ba(OH)_2_·xH_2_O, Y(NO_3_)_3_·6H_2_O, (CH_3_CO_2_)_3_Er, Yb(NO_3_)_3,_ and NaBF_4_ were put into a Teflon cup. Oleic acid and ethanol were added into the mixture for corresponding to a certain proportion. The pH value of the mixed solution was adjusted to 9 by using NH_3_·H_2_O. After magnetic stirring for 30 min, the Teflon cup was held into stainless steel sealing autoclave and heated to 200 °C for 16 h. When the autoclave was naturally cooled to the room temperature, the product was centrifuged by ethanol and deionized water for three times respectively and dried at 60 °C for 12 h.

### Characterization

X-ray diffraction (XRD) was obtained on BrukerD8 advance at a scanning speed of 10°/min in the 2*θ* range from 10 to 70 with Cu Kα radiation. Photoluminescence spectroscopy (PL) was recorded on a fluorescence spectrometer (FLS920, Edinburgh Instruments) upon continuous wave excitation of 980-nm laser diode. Scanning electron microscope (SEM) and energy dispersive spectrometer (EDS) were recorded on S-3400N-II.

## Results and Discussion

**Fig. 1:**
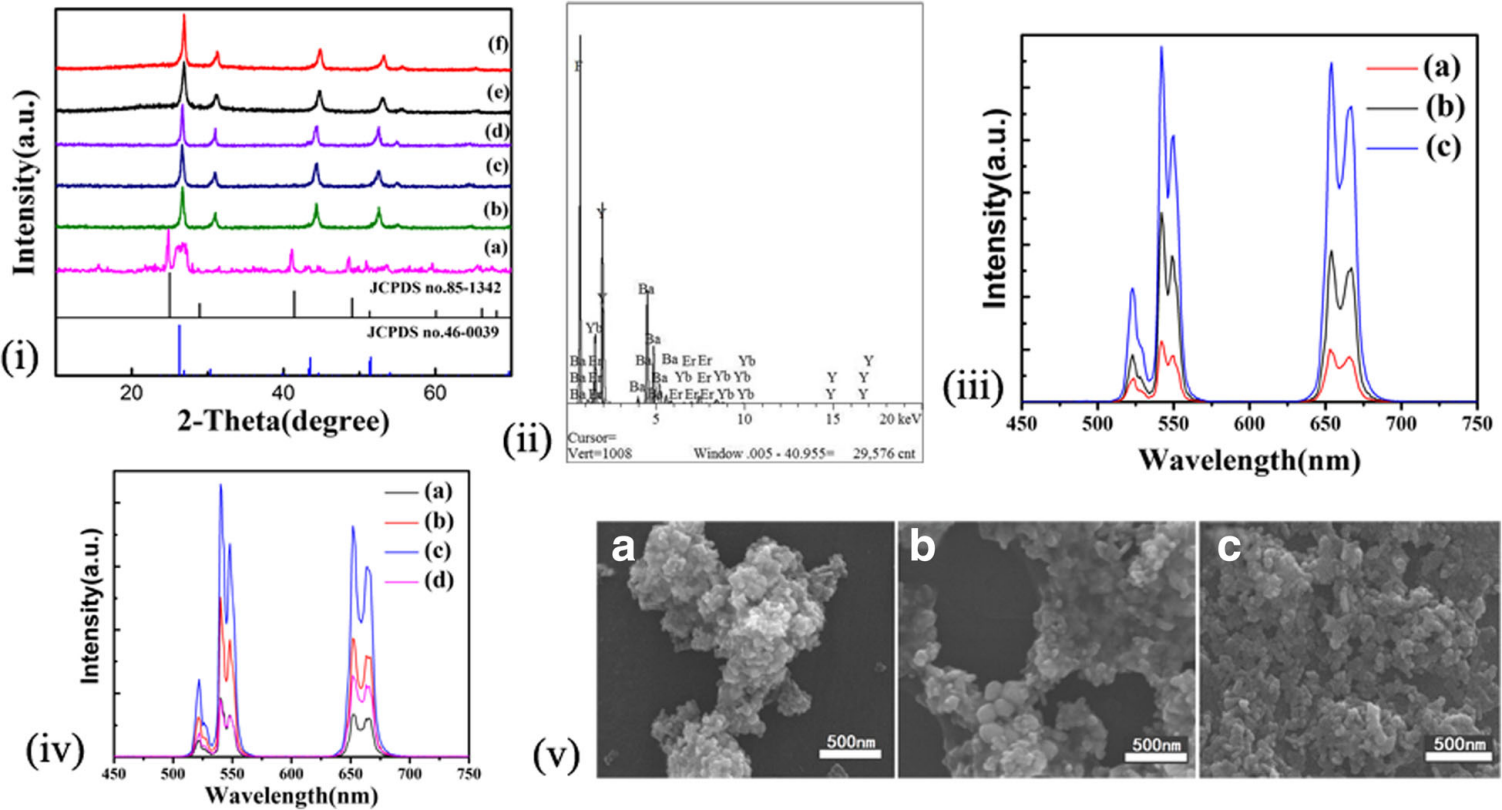
**i** XRD patterns of the prepared 2%Er^3+^, 20%Yb^3+^-codoped BaYF_5_, pH is equal to 9 except (a), whose pH equals 4. (a) 200 °C, 16 h. (b) 200 °C, 12 h. (c) 200 °C, 16 h. (d) 200 °C, 24 h. (e) 180 °C, 16 h. (f) 220 °C, 16 h. The standard XRD pattern of BaYF_5_ (JCPDS no.46-0039) and BaF_2_ (JCPDS no.85-1342) is also given for comparison. **ii** EDS of the product corresponding to XRD (d). **iii** UC emission spectra of the samples synthesized at 200 °C, (a) 12 h, (b) 16 h, (c) 24 h. **iv** UC emission spectra of the products synthesized for 16 h. (a) 180 °C, pH = 9. (b) 200 °C, pH = 9. (c) 220 °C, pH = 9. (d) 200 °C, pH = 4. **v** SEM images of the prepared BaYF_5_ synthesized under different conditions. (A) 200 °C, 16 h. (B) 220 °C, 16 h. (C) 200 °C, 24 h

Figure 1i presents the XRD patterns of the BaYF_5_:20%Yb^3+^, 2%Er^3+^ synthesized by different conditions. The diffraction peaks of all the samples can be readily indexed to the standard tetragonal-phase BaYF_5_ (JCPDS no.46-0039) except for Fig. 1i (a) owing to the generation of extra phase BaF_2_ at the pH value of 4_._ When the pH increased from 4 to 9, the crystalline of the sample was enhanced. In the meanwhile, the BaF_2_ phase disappeared as well. There was no extra peaks of other phase appeared, revealing that the varying experimental conditions have little influence on the crystal structure of the sample. It is noteworthy that all the diffraction peaks are shifted to higher 2*θ* side, which indicates the lattice constant becomes smaller because the radii of Er^3+^ or Yb^3+^ are smaller than those of Y^3+^ [25, 26]. Furthermore, it is easy to find the rules that when the reaction time increases, the intensity of the diffraction peaks is simultaneously enhanced. Similar conclusions are drawn when temperatures rise. It concludes that the above reaction conditions can promote the growth of BaYF_5_ crystals. EDS spectrum analysis of a specific sample was indicated in Fig. 1ii. As shown on the diagram, the presence of the elements of Ba, Y, F, Yb, and Er in the given sample was confirmed. According to the XRD and EDS results, Er^3+^ and Yb^3+^ were successfully doped into BaYF_5_. Figure 1v shows the SEM images of BaYF_5_ synthesized under different conditions. The as-prepared samples shown in Fig. 1v (A) are microspheres with a size of approximately 45 nm. However, they are not finely dispersed and aggregated to some extent. According to its XRD diagram in Fig. 1i (c), the size of the crystal can be roughly calculated by Scherrer’s equation:

$$ D= K\gamma /B\ \cos \theta $$

where *K* is Scherrer constant (*K* equals 0.89), *γ* is the X-ray wavelength (*γ* equals 0.15405 nm), *B* is the full width at half maximum of diffraction peaks of samples, and *θ* is the diffraction angle of the observed peak [27, 28]. The strongest intensity of diffraction peak at 2*θ* = 26.689° was used to calculate the average size of crystal. The average size of crystal is estimated to be 41.7 nm which is closed to the size (45 nm) by observation of the SEM chart. As represented in Fig. 1v (B), when the reaction temperature was increased to 220 °C, the dispersions of the particles became relatively high. However, the size of the crystal was uneven and some larger particles with size of approximately 180 nm appeared. When the reaction time was prolonged to 24 h, the nanocrystals are relatively well dispersed with uniform particle morphology. The sizes are about 30 nm which are basically consistent with the estimates (24.9 nm) from the XRD data. Figure 1iii, iv shows the UC luminescence spectra of BaYF_5_:Er^3+^/Yb^3+^ synthesized via different experimental conditions under excitation at 980 nm. The main emission band of Er^3+^ is 520, 540, and 654 nm as a result of ^2^H_11/2_ → ^4^I_15/2_ (green), ^4^S_3/2_ → ^4^I_15/2_ (green), and ^4^F_9/2_ → ^4^I_15/2_ (red) transitions, respectively. In Fig. 1iii, iv, as the temperature increases, it is beneficial to the crystal growth of the product, while prolonging the reaction time, increasing pH has the same effect. The UC luminescence intensity can be enhanced due to the formation of higher crystalline. When the reaction time was prolonged, or pH was adjusted from 4 to 9, the nanoparticles have better crystalline owing to their higher dispersions and more uniform sizes.

**Fig. 2:**
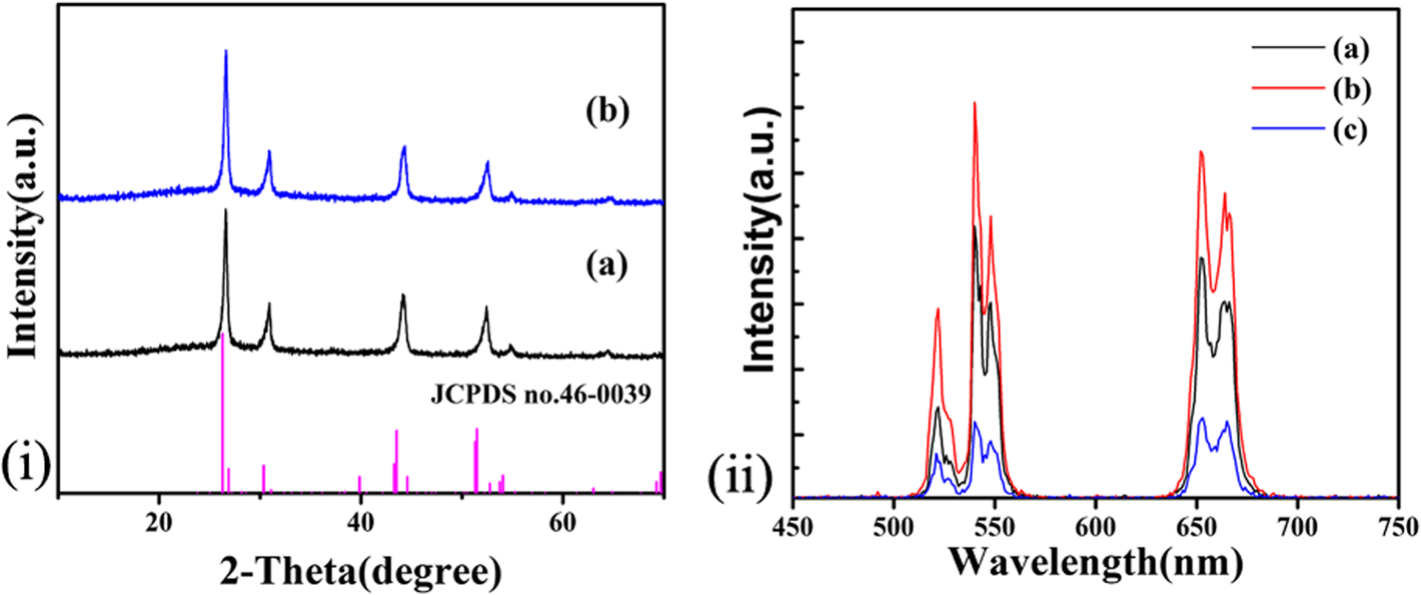
**i** XRD patterns of the prepared 2%Er^3+^, Yb^3+^-codoped BaYF_5_ synthesized at 200 °C for 16 h with different Yb^3+^ concentrations, (a) 10%Yb^3+^ and (b) 30%Yb^3+^, the standard XRD pattern of BaYF_5_ (JCPDS no.46-0039) is also given for comparison. **ii** UC emission spectra of the samples synthesized at 200 °C for 16 h with different Yb^3+^ concentrations. (a) 10%Yb^3+^, (b) 20%Yb^3+^, (c) 30%Yb^3+^

Figure 2i demonstrates the XRD patterns of the BaYF_5_: *x*Yb, 2%Er (*x* = 10%, 30%). All the diffraction peaks are perfectly matched with the standard pattern of BaYF_5_ crystals (JCPDS no.46-0039). It shows that the doping of rare earth ions does not affect the crystal growth. As shown in Fig. 2ii, when the Yb^3+^ concentration increases from 10 to 20%, the UC luminescence intensity rapidly rises until the Yb^3+^ concentration exceeds 20% on account of the concentration quenching. It is concluded that the 20% concentration is the optimum concentration.

**Fig. 3:**
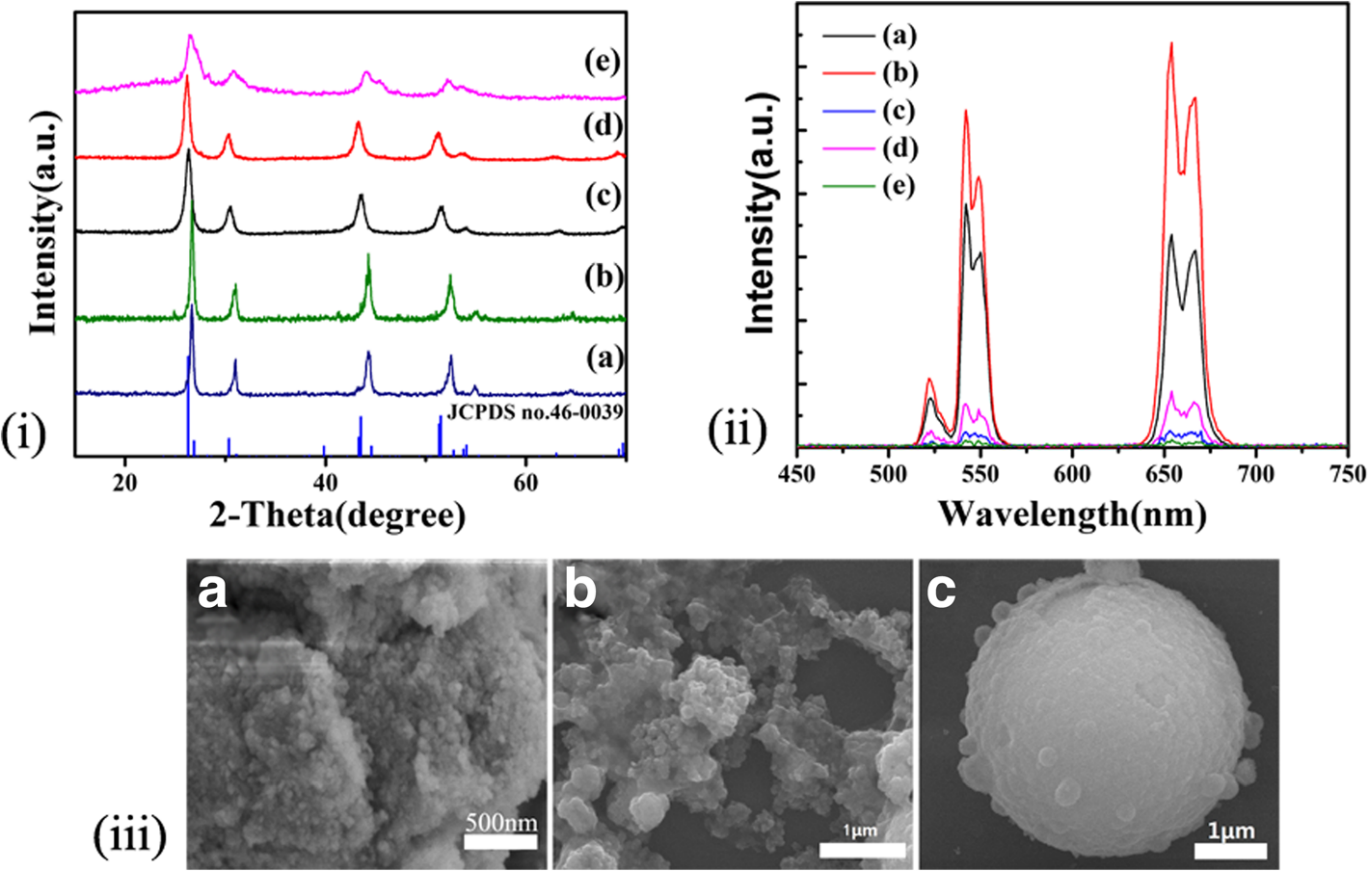
**i** XRD patterns of the prepared 2%Er^3+^, 20%Yb^3+^-codoped BaYF_5_ synthesized at 200 °C for 24 h, (a) solvent is ethanol, (b) solvent consists of 95% ethanol and 5% PEI, (c)–(e) citric acid as surfactant was added, the ratio of CIT to Y is 1:1, 2:1, and 4:1, respectively. The standard XRD pattern of BaYF_5_ (JCPDS no.46-0039) is also given for conducting the comparison. **ii** UC emission spectra of BaYF_5_ prepared. (a) Solvent is ethanol, (b) solvent consists of 95% ethanol and 5% PEI, (c) CIT/Y = 1:1, (d) CIT/Y = 2:1, (e) CIT/Y = 4:1. **iii** SEM images of the samples synthesized via adding different surfactants with different concentrations. (A) 5% PEI, (B) CIT/Y = 1:1, (C) CIT/Y = 4:1

Figure 3i exhibits the XRD patterns of the BaYF_5_:Yb^3+^/Er^3+^ nanocrystals obtained by adding different surfactants. All the diffraction peaks are perfectly matched with the standard card tetragonal-phase BaYF_5_ (JCPDS no.46-0039). When 5% polyetherimide (PEI) was added, the intensity of the diffraction peaks is enhanced, which indicates that PEI can promote the growth of BaYF_5_ crystals. Moreover, after the addition of citric acid, the diffraction peaks are shifted to lower angle. This proves that when citrate (CIT) was added, the cell volume of the sample becomes larger gradually. The other reason may be that citric acid is covered on the crystal surface, rare earth ions are hard to dope into the host lattices. Besides, the diffraction peaks become different from the others with a little flaw as the CIT/Y = 4:1. The conceivable reason lies in the high CIT concentration leading to the BaYF_5_ unit cell parameter change and lattice distortion. As illustrated in Fig. 3iii (A), when 5% PEI was added in ethanol, nanocrystals became massive clumps which consist of a large number of spherical particles with narrow size distribution. Figure 3iii (B) and (C) shows that when the surfactant with a concentration of CIT/Y = 1:1 was added, the overall size of crystal relatively became larger. As can be seen from the diagram, the sample tends to aggregate without obvious boundaries in some areas. As the surfactant concentration ratio rises to 4:1, the maximum size of the particles increases to 4 um with the surface covered by some other smaller spherical particles. As the surfactant concentration increases, the CIT coverage capacity is enhanced [29], leading to the formation of crystal clusters. As shown in Fig. 3ii, both green emission and red emission are enhanced after adding 5% PEI in ethanol. The long-chain amino groups of PEI can form the complex structures with metal ions by coordination. PEI can inhibit particle growth by tightly wrapping on the surface to improve the crystalline. On the contrary, after adding citric acid, the UC luminescence emission decreased greatly owing to the enlargement of crystal size and the decline of rare earth ion content.

**Fig. 4:**
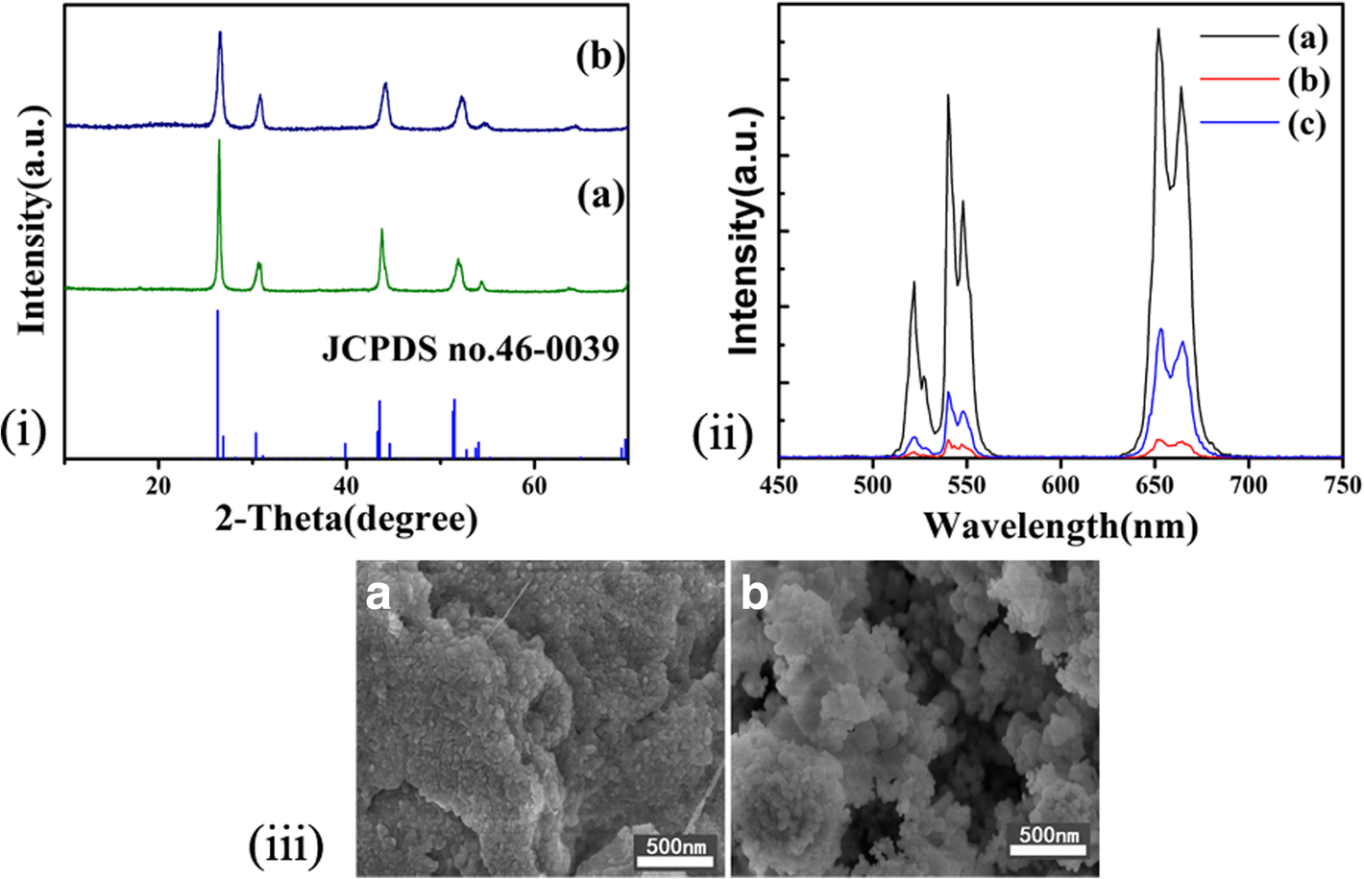
**i** XRD patterns of the prepared 2%Er^3+^, 20%Yb^3+^-codoped BaYF_5_ synthesized at 200 °C for 24 h; 5% PEI was added for conducting convenient comparison. (a) and (b) fluoride source was NH_4_F and NaF, respectively. The standard XRD pattern of BaYF_5_ (JCPDS no.46-0039) is also given for conducting the comparison. **ii** UC emission spectra of samples. (a) NaBF_4_. (b) NH_4_F. (c) NaF. **iii** SEM images of the products (A) NH_4_F. (B) NaF

Figure 4i shows the XRD pattern of products obtained from different fluoride sources. There are no peaks of impurities appear, demonstrating that the change of fluoride sources does not affect the crystallization of BaYF_5_. It is worth noting that there are fewer shifts of the diffraction peaks of samples obtained from NH_4_F or NaF than those of samples obtained from NaBF_4_. This indicates that NH_4_F and NaF released F^−^ disorderly and rapidly, resulting in the difficulty of the control synthesis of crystals [30]. As a consequence, the rare earth ions become difficult to enter into the host lattices. Figure 4iii represents the SEM images of the sample using NH_4_F and NaF as fluoride sources. The particles are similar to those nanocrystals synthesized by adding 5% PEI. However, the shapes are more irregular relative to those obtained from NaBF_4_. As can be seen from Fig. 4ii, the sample which used NaBF_4_ as fluoride source shows the highest UC emission efficiency owing to the benefits of the crystal growth generating a uniform sphere shape. Particles in smaller sizes will have more Er^3+^ on the submicron surface, causing more surface vibrations for conducting acceleration in red and green emission. Moreover, the distance among Er^3+^ becomes smaller and cross relaxation happens (^2^H_11/2_ + ^4^I_15/2_ → ^4^I_9/2_ + ^4^I_13/2_). As a result, the green band (^2^H_11/2_, ^4^S_3/2_ → ^4^I_15/2_) becomes easy to quench in smaller sizes, but the red band (^4^F_9/2_-^4^I_15/2_) becomes more difficult to quench [24, 31].

**Fig. 5:**
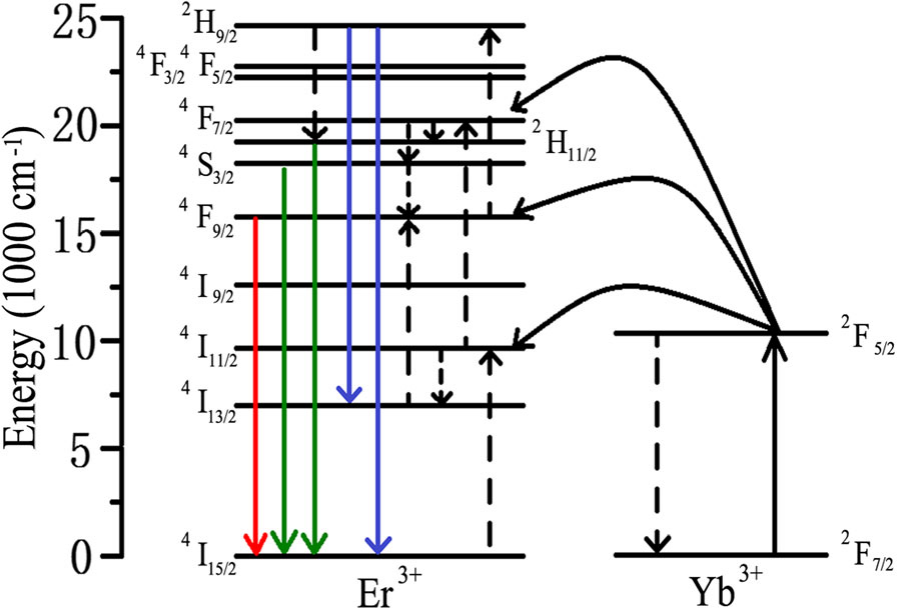
Schematic diagram of energy levels between Er^3+^ and Yb^3+^

Figure 5 demonstrates the schematic energy levels of Yb^3+^ and Er^3+^. In the meanwhile, it depicts the UC luminescence process mechanisms explaining the generation of green and red emissions under 980-nm laser excitation. In the Yb^3+^/Er^3+^-codoped BaYF_5_ system, via absorbing the first 980-nm photon, Yb^3+^ ion in the ^2^F_7/2_ ground state transfers to the excited state ^2^F_5/2_. When it goes back to the ground state, the energy is transferred to Er^3+^ ion to populate the ^4^I_11/2_ state. The second 980-nm photon, or energy transfer from another excited Yb^3+^, can then pump Er^3+^ ion into ^4^F_7/2_ level. The lower energy states ^2^H_11/2_ and ^4^S_3/2_ can be populated by nonradiatively decaying ^4^F_7/2_ state. The transmissions of the electron from ^2^H_11/2_ and ^4^S_3/2_ to the ^4^I_15/2_ ground state emits green emissions. Alternatively, Er^3+^ ion in the ^4^I_11/2_ state may also nonradiatively relax to ^4^I_13/2_ state. ^4^F_9/2_ state of Er^3+^ can be populated by absorption of photon or energy transfer from Yb^3+^. The UC red emissions occur through the transition of ^4^F_9/2_ to ^4^I_15/2_. Some electrons in the ^4^F_9/2_ level may be excited to ^2^H_9/2_ via a phonon-assisted energy transfer process, and blue emissions can be observed. The emission bands at 520, 540, and 654 nm could be corresponding to electron transfer from the excited level ^2^H_11/2_, ^4^S_3/2_, and ^4^F_9/2_ to the ground state ^4^I_15/2_ of Er^3+^, respectively [19, 32, 33].

## Conclusion

In summary, BaYF_5_:20%Yb^3+^, 2%Er^3+^ have been successfully synthesized via a convenient solvothermal method. It is found that the use of NaBF_4_ as a fluoride source or adding 5% PEI as surfactant can effectively improve the crystalline and particle dispersion which can promote the UC emission. Compared with PEI, as CIT concentration raised, the nanoparticles gradually become larger, which is inversely proportional to luminous properties. It is obvious that nanocrystals via 220 °C of heat treatment temperature for 24 h are an optimum reaction condition of the excellent luminescence properties. These behaviors might be attributed to their great uniform sizes, well dispersing, and high crystalline.
